# The Immunomodulatory Role of Gemcitabine in Triple Negative Breast Cancer

**DOI:** 10.3390/cells14201604

**Published:** 2025-10-16

**Authors:** Cory Fines, Syed Umbreen, Elaine Gilmore, Helen McCarthy, Niamh Buckley

**Affiliations:** Medical Biology Centre, School of Pharmacy, Queen’s University Belfast, 97 Lisburn Road, Belfast BT9 7BL, Northern Ireland, UK; s.umbreen@qub.ac.uk (S.U.);

**Keywords:** triple negative breast cancer, immunotheerapy, chemotherapy, gemcitabine

## Abstract

Triple negative breast cancer (TNBC), defined for its lack of expression/amplification of three major receptors, makes up ~15% of all BC cases but a majority of all BC deaths. TNBC has been found to be the most immune-rich among BC subtypes, and progress has been made in the development of immunotherapies; however, not all patients are eligible, and response can be limited. Therefore, there is a significant clinical need to enhance the response to these treatments. Given chemotherapy is the core component of TNBC treatment, and is given in combination with immunotherapy, its potential immunomodulatory impact warrants exploration. Gemcitabine, currently used for the treatment of metastatic TNBC, has been reported to have potential immunomodulatory properties that create a more immune-favourable TME for combination with immunotherapies and/or improved outcome. We therefore investigated the use of gemcitabine as an immunomodulator in a primary 4T1 TNBC mouse model. Gemcitabine was able to reduce pro-tumour immune cells including macrophages and MDSCs while increasing T-cell abundance, therefore resulting in a less immunosuppressive TME. We demonstrated that this immune response was both temporal and dose-dependent, which has impact for planning and scheduling combination treatments. In conclusion, we have demonstrated that gemcitabine modulates the TME in ways that could not only enhance the direct anti-tumour effects of gemcitabine itself but also potentially enhance responsiveness to immunotherapy. This work has laid the foundation for further studies investigating combination therapy for the treatment of TNBC.

## 1. Introduction

Breast cancer (BC) is the most diagnosed cancer and the second leading cause of cancer-related death in women [1]. Triple negative breast cancer (TNBC), accounting for approximately 15% of all BC cases, is characterised by the absence of the oestrogen receptor (ER), progesterone receptor (PR) and lack of amplification of the human epidermal growth factor receptor 2 (HER2) [2]. This renders TNBC unresponsive to hormone therapies and HER2-directed treatments, which have significantly improved outcomes for other breast cancer subtypes. Therefore, chemotherapy remains the backbone of the systemic standard of care for TNBC [3].

The tumour microenvironment (TME) has been shown to play a critical role in tumour progression and survival. Furthermore, the TME can influence treatment response such as resistance to chemotherapy [4]. Therefore, knowledge of the makeup of the patients’ TME can be used help guide the best treatment strategy for that individual [5]. While, historically, BC has been seen as immune “cold”, TNBC is the most immunogenic BC subtype, with increased tumour-infiltrating lymphocytes (TILs) correlating with improved prognosis and sensitivity to immunotherapy [6,7]. However, immunosuppressive mechanisms are often upregulated in TNBC, which negatively impacts overall TIL function and recruitment [8]. This has led to the use of immunotherapies such as the programmed cell death protein 1 (PD-1) checkpoint inhibitor, pembrolizumab, which reinstates cytotoxic T-cell function [9].

While immunotherapies are now being used in the clinic, they are limited to a small proportion of the patient population, with only 20–60% of TNBC tumours being programmed death ligand 1 (PD-L1)-positive [10,11,12,13,14]. Furthermore, they show limited effect (~22% objective response rate) in metastatic TNBC (mTNBC) patients [15]; therefore, there is an ongoing need to improve response and/or identify alternate options. It has been shown that chemotherapy alone can have an immunomodulatory role on the composition of the TME, which could enable new combination therapy regimes, although this varies between different chemotherapies and can modulate both pro- and anti-tumourigenic immune populations [16]. Gemcitabine (GEM) is an anti-metabolite chemotherapy used frontline for pancreatic cancer, as well as used in combination with other chemotherapies for mTNBC [17]. Gemcitabine has been reported to reduce pro-tumourigenic cells such as T-regulatory cells (T-regs) and monocytic-derived suppressor cells (MDSCs) in pancreatic cancer patients [18]. A limited number of animal models have also shown similar effects in TNBC; however, the extent of immune modulation by GEM in TNBC is still not well understood [19,20]. Throughout this paper, we explore the pharmacodynamic immune properties of gemcitabine including its temporal profile, minimum effective (immune) dosage and route of administration for the treatment of TNBC. Our work builds and expands on past work by further uncovering pharmacodynamic properties of GEM while starting to delineate the mechanism by which it influences immune cells. Having a better understanding of how GEM influences the immune system, both systemically and in the TME, allows for better tailoring of treatment, especially when combining with other immunotherapies in the future.

## 2. Materials and Methods

### 2.1. Cell Lines

4T1 and THP1 cells were maintained in RPMI-1640 +L-glutamine (Thermo Fisher, Oxford, UK, Gibco #11875-093). Media was supplemented with 10% foetal bovine serum (FBS, Thermo Fisher, Oxford, UK) and cells were grown at 37 °C with 5% CO_2_. Cells were all obtained from ATCC and routinely tested for mycoplasma.

### 2.2. Macrophage Polarisation

THP1 cells were seeded at 1 × 10^6^ cells per p60 dish in the presence of phorbol 12-myristate 13-acetate (PMA, Thermo Fisher, Oxford, UK, J63916, 5 ng/mL). After 24 h media was replaced and the now differentiated THP1 cells (D-THP1) were incubated for 72 h before the addition of cytokines for 24 h to induce polarisation. IL4 (PeproTech, Cheshire, UK, 200-04, 30 ng/mL) or interferon gamma (IFNγ) (Proteintech, Manchester, UK, HZ-1301, 20 ng/mL) in combination with lipopolysaccharides (LPS) (LPS, Sigma, Leicestershire, UK, L5293,250 ng/mL) were used for M2-like and M1-like polarisation, respectively. For analysis of GEM’s direct role on macrophage polarisation, Gemcitabine Hydrochloride (GEM) (Fluorochem, Derbyshire, UK) (0.1 mM) was added to the D-THP1 cells for 24 h and compared to untreated D-THP1 cells. For analysis of GEM’s indirect role on macrophage polarisation, GEM (0.1 mM) was added onto 4T1 cells for 48 h. This condition media was transferred to D-THP1 and left for 24 h and compared to untreated 4T1-conditioned media.

### 2.3. mRNA Extraction and PCR

Cells were collected in TriPure (Roche, Switzerland) and mRNA was extracted according to the manufacturer’s recommendation. mRNA was converted to cDNA using the Transcriptor First Strand cDNA synthesis kit (Roche, Hertfordshire, UK, #04379012001) and subsequently used for real-time PCR, performed using the LightCycler 480 SYBR Green I Master system (Roche, Germany), with gene expression normalised to the housekeeper gene b-actin. All primers are listed in Supplementary Table S1.

### 2.4. Macrophage Chemotaxis

4T1 cells were seeded at 2.5 × 10^5^ cells per p60. After 24 h, cells were treated with GEM (0.1 mM). After 48 h, media was collected and transferred to a 24-well plate. A transwell filter (8 mm) was placed in each well with either 2 × 10^5^ THP1 or D-THP1 cells. After 72 h, THP1 cells from the bottom of the 24-well were counted. D-THP1 transwell was removed and washed with PBS and the inside of the transwell was cleaned with a cotton swab. The transwell was then fixed with 70% ethanol for 20 min and then stained with crystal violet. The inside of the transwell was cleaned with a cotton swab and the crystal violet was reabsorbed with a 1:1 ratio of 100% ethanol and 0.2 M Sodium Citrate. Absorbance was read at 570 nm using a FLUOstar Omega plate reader (BMG Labtech, Bucks, UK).

### 2.5. Cell Viability

4T1 cells were seeded at 4 × 10^4^ cell per 24-well and left to incubate for 24 h before treatment with GEM for 72 h at a range of concentrations (0–10 mM). After 72 h, Resazurin (Fluorochem) (100 mM) was added and incubated for 2 h, before reading absorption at 570 nm using a FLUOstar Omega plate reader (BMG Labtech, Bucks, UK). Data was presented as percentage viability relative to control.

### 2.6. Colony Formation Assay

4T1 cells were seeded in a 24-well plate and treated with GEM, as previously described. Then, 24 h post-treatment, cells were reseeded at 300 cells per well of a 6-well plate. Cells were left to incubate for 14 days before staining with crystal violet. Plates were imaged and colonies were counted using ImageJ 1.53t.

### 2.7. Establishment of a 3D Spheroid Model

4T1 cells were seeded at 500 cells per well in 96-well round bottom plates and left to incubate for 24 h. After 24 h, 5% Matrigel (Corning) was added to achieve a working concentration of 2.5%. A total of 48 h later (Day 4), cells were treated with 0–10 mM GEM, and spheroid volumes were measured using a plate imager every day for 2 weeks. The increase in spheroid volume was plotted and the change in growth rate after treatment was seen as an indirect measure of cell viability change.

### 2.8. In Vivo

All experiments were compliant with the UK Scientific Act of 1986 and performed under project license 2881. Six- to seven-week-old female Balb/C mice were purchased from Charles River Laboratories (Ireland, UK) and left for 7–10 days for adaption. 4T1 (5 × 10^4^) cells were injected into the 4th mammary fat pad under light anaesthesia. Body weight and tumour volume were measured three times per week, and tumour volume was calculated using GMD (Equation (1)). Based on pilot growth kinetic studies, by Day 16, tumours were measurable averaging~65–70 mm^3^ which was at sufficient size to allow robust tumour measurements and ex vivo analysis. Due to the reproducibility of the experiments, all experiments began on Day 16, at which time mice were randomised into the different treatment groups (*n* = 3–7 per group) according to tumour volume, ensuring mean starting values did not vary significantly across the groups. GEM was reconstituted in PBS and was administered at a range of doses (60, 30 and 15 mg/kg) into the intraperitoneal (I.P.) cavity, as well as at 20 mg/kg intravenously (I.V.). Up to 3 treatments were administered on Days 16, 21 and 26.

(1) $$r=\frac{1}{2}\sqrt{\left(length\times width\right)}\;\;Volume=\frac{4}{3}\pi \;r^{3}$$

### 2.9. Flow Cytometry

Tumours, spleen and blood were collected from mice at 24, 48, 72 or 96 h post-treatment. Tumours were digested at 37 °C for 30 min using collagenase A (IV) (Roche, Hertfordshire, UK, #10103578001). Tumour suspensions were filtered through a 100 µm filter before centrifugation at 2000 rpm. The resulting cell pellet was resuspended in PBS and stored on ice. Spleens were mechanically dissociated and filtered through a 100 µm filter. The spleen cell suspension was centrifuged at 2000 rpm, and the resulting pellets were resuspended in red blood cell lysis buffer (Invitrogen, Cheshire, UK, #00-4333-47) and incubated for 4 min on ice before stopping the reaction with an equal volume of cold PBS. Cells were pelleted by centrifugation at 2000 rpm and resuspended in ice-cold PBS. Spleen and tumour cell samples were counted, and 1 × 10^6^ cells were seeded in an FCS tube. Cells were blocked with FC block (Supplementary Table S2) for 15 min at 4 °C. Blood was collected in a heparin-coated tube and 200 µL was transferred to an FCS tube. All FCS samples were stained with the viability dye and antibodies for 30 min at room temperature (Supplementary Table S2). Tumour and spleen samples were fixed following the manufacturer’s protocol (Biolegend, Cambridge, UK, #424401), washed and resuspended in PBS. Blood was resuspended in RBC lyse and fix buffer (Invitrogen, Cheshire, UK, #2781577), washed and resuspended in PBS. Samples were analysed using a BD FACSymphony A5 flow cytometer. Single-stained beads (Invitrogen, Cheshire, UK, 01-3333-42, A10346) were run for compensation. Gates were made with the use of fluorescence-minus-one (FMO) controls for each fluorophore. All data was analysed using FlowJo, with gating strategies found in Supplementary Figure S1.

### 2.10. RNASeq

For eight mouse samples, total RNA was extracted using RNAlater and subsequently cleaned using the Zymo Research RNA Clean & Concentrator kit with DNase treatment. RNA from these samples was quantified using the NanoDrop One and quality assessed on the Agilent TapeStation 4200 RNA ScreenTape Assay.

RNASeq libraries were prepared using the KAPA RNA HyperPrep Kit with RiboErase (Roche, Hertfordshire, UK, ) and KAPA Universal Adapters. Libraries were then amplified with KAPA HiFi HotStart ReadyMix with quality control performed using the Qubit dsDNA High Sensitivity Assay Kit (Thermo Fisher, Oxford, UK). The pooled library was sequenced on an Illumina NextSeq 2000 platform generating 50 bp paired-end reads.

### 2.11. RNASeq Analysis

RNASeq data from mouse samples were processed using a standardised pipeline. Raw sequencing reads were first assessed for quality with FastQC v0.11.8, followed by alignment to the reference genome—GRCm38.p5_GENCODE.vM15—using STAR v2.7.3a. Gene-level quantification was performed with HTSeq v0.11.3 using corresponding GTF annotation files (gencode.vM15.annotation.gtf). Alignment and library quality metrics were further evaluated using Picard v3.0.0 and Qualimap v2.3.0, and a comprehensive summary of all metrics was generated with MultiQC v1.14. Differential gene expression was carried out using DeSeq2 in R. Gene set enrichment analysis (GSEA) was performed using the software downloaded from www.gsea-msigdb.org using the Hallmarks gene set [21,22]. The TIMER2 platform (http://timer.cistrome.org, accessed 17 August 2025) [23] was used to estimate immune populations using the TIMER [24].

### 2.12. Statistical Analysis

Statistical analysis was performed using GraphPad Prism v10 (GraphPad Software). For comparing two populations, a nonparametric unpaired Mann–Whitney U-test was performed. For comparing more than two populations, a nonparametric Kruskal–Wallis test was performed with post hoc Dunn’s correction for multiple testing. Data is presented as mean ± SEM, where each symbol represents one animal for all in vivo work, or one replicate in all in vitro work. Flow cytometry data for each immune population was combined when possible and reported as percentage change. The treated group values were normalised to the average of the untreated group within each individual experiment before combining. * *p* < 0.05, ** *p* < 0.01, *** *p* < 0.001, **** *p* < 0.0001. A ROUT test (Q = 10%) was performed for outliers and removed when needed (*n* = 0–3). Flow cytometry plots were made with FlowJo v10 (FlowJo, LLC; USA), graphs with GraphPad Prism and R, and experimental illustrations with Biorender (BioRender.com).

## 3. Results

### 3.1. The Immunomodulatory Role of Gemcitabine Is Temporally Dependent

**Figure 1:**
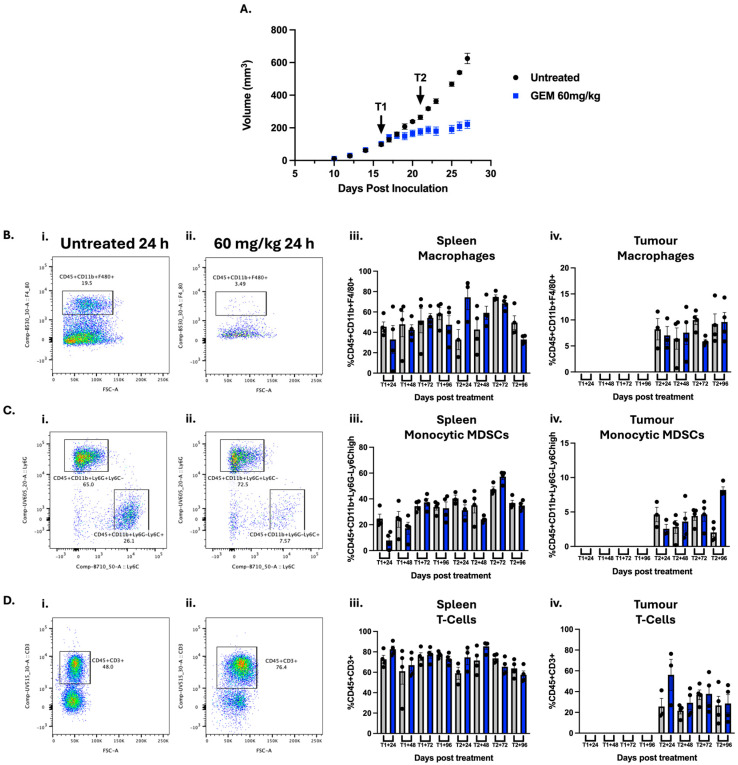
The temporal immune profile of gemcitabine in 4T1 tumour-bearing mice: 5 × 10^4^ 4T1 cells were inoculated into the 4th mammary fat pad of female Balb/C mice (Day 0). Mice were treated twice with 60 mg/kg GEM on Days 16 (Treatment1 (T1)) and 21 (Treatment2 (T2)) after cell inoculation. (**A**) Tumour growth was monitored throughout the study. Tumours and spleen were collected 24-96 h after treatment 1 and 2 (T1/2 + 24/48/72/96) and analysed via flow cytometry. Representative gating of (**B**) macrophages (CD45^+^CD11b^+^F4/80^+^), (**C**) monocytic myeloid-derived suppressor cells (M-MDSCs; CD45^+^CD11b^+^Ly6G^−^Ly6C^+^) and (**D**) T-cells (CD45^+^CD3^+^) in spleen following (**i**) saline or (**ii**) gemcitabine treatment, with quantification of macrophage abundance in the (**iii**) spleen and (**iv**) tumour over time.

The first immune pharmacodynamic property investigated was the temporal profile of GEM. Following the literature, 4T1 tumour-bearing mice were treated twice with 60 mg/kg GEM via I.P. administration [19], with samples collected 24–96 h after the first (Day 16—T1) and second treatment (Day 21—T2) of GEM and processed for flow cytometry (Figure 1). Consistent with previous studies [19,20], tumour volume and spleen mass decreased following treatment (Figure 1A and Figure S2D).

In order to assess any potential changes in immune populations, we designed a multiplex panel allowing interrogation of the T-cell (CD45^+^CD3^+^), T-helper (CD45^+^CD3^+^CD4^+^CD8^−^), cytotoxic T-cell (CD45^+^CD3^+^CD4^-^CD8^+^), macrophage (CD45^+^CD11b^+^F480^+^), monocytic (M-) MDSCs (CD45^+^CD11b^+^Ly6G^−^Ly6C^high^), granulocytic (G-) MDSCs (CD45^+^CD11b^+^Ly6G^+^Ly6C^−^) and pan-MDSCs (CD45^+^CD11b^+^GR1^+^) immune populations. These were chosen based on clinical relevance and previous reports of modulation by GEM [20]. In untreated mice, we observed a significant positive correlation between spleen mass and tumour volume (Figure S2A). This increase in spleen mass also correlated with decreased splenic abundance of T-cells, and an increased abundance of M-MDSCs (Figure S2B,C).

Following treatment, spleen mass decreased, and an increase in T-cells with concomitant decrease in MDSCs and macrophages was observed in the spleen (Figure 1 and Figure S2). While a change in overall T-abundance was seen, there was no measurable change in helper or cytotoxic T-cell populations (Figure S4). Furthermore, there were no measurable changes in splenic G-MDSCs or pan-MDSCs (Figure S4). The greatest reduction in spleen mass was at 24 h post-treatment (after either one or two treatments) (Figure S2D), which correlated with the greatest increase in T-cells and reduction in MDSCs and macrophages (Figure 1B–D and Figure S2). 48 h post-treatment, the populations of immune cells returned to untreated baseline abundance, with spleen mass also recovering in a time-dependent manner, returning to baseline by 96 h post-treatment (Figure 1 and Figure S2D).

This inverse correlation of T-cells and MDSCs has also been reported in blood and tumours extracted from 4T1 tumour-bearing mice [25]. In this study, we also report an increase in T-cells and decrease in MDSCs in the tumour (Figure 1) and blood (Figure S3A,B). We were unable to accurately detect immune populations in the tumour after treatment #1, possibly due to lower overall abundance. After treatment #2, an increase in total T-cells and a reduction in macrophages and M-MDSCs was observed in the tumour (Figure 1(iv)). As seen in the spleen, these changes were greatest 24 h after treatment and diminished over time. Again, there were no measurable changes in the tumour helper or cytotoxic T-cell populations or the G- or pan-MDSCs.

While preparing the manuscript, Kitelinger et al. reported immune population changes 24, 48 and 96 h after a third treatment of GEM (60 mg/kg I.P.) at Day 26 in a 4T1 model [20]. To ensure the same trends were seen after this third treatment, an additional time point was assessed and similar decreases in M-MDSCS and increase in T-cells after treatment 3 were observed, as with the other two treatments. (Figure S3C–F). To retain a tumour that could encompass all immune populations, neither undetectable due to too small of size nor influenced by necrotic/hypoxic regions found at a larger size, all subsequent studies were performed after treatment #2 [26,27,28,29,30,31]. Furthermore, all tissues were taken 24 h after treatment to see the greatest immune impact.

### 3.2. The Immunomodulatory Role of Gemcitabine Is Dose-Dependent

**Figure 2:**
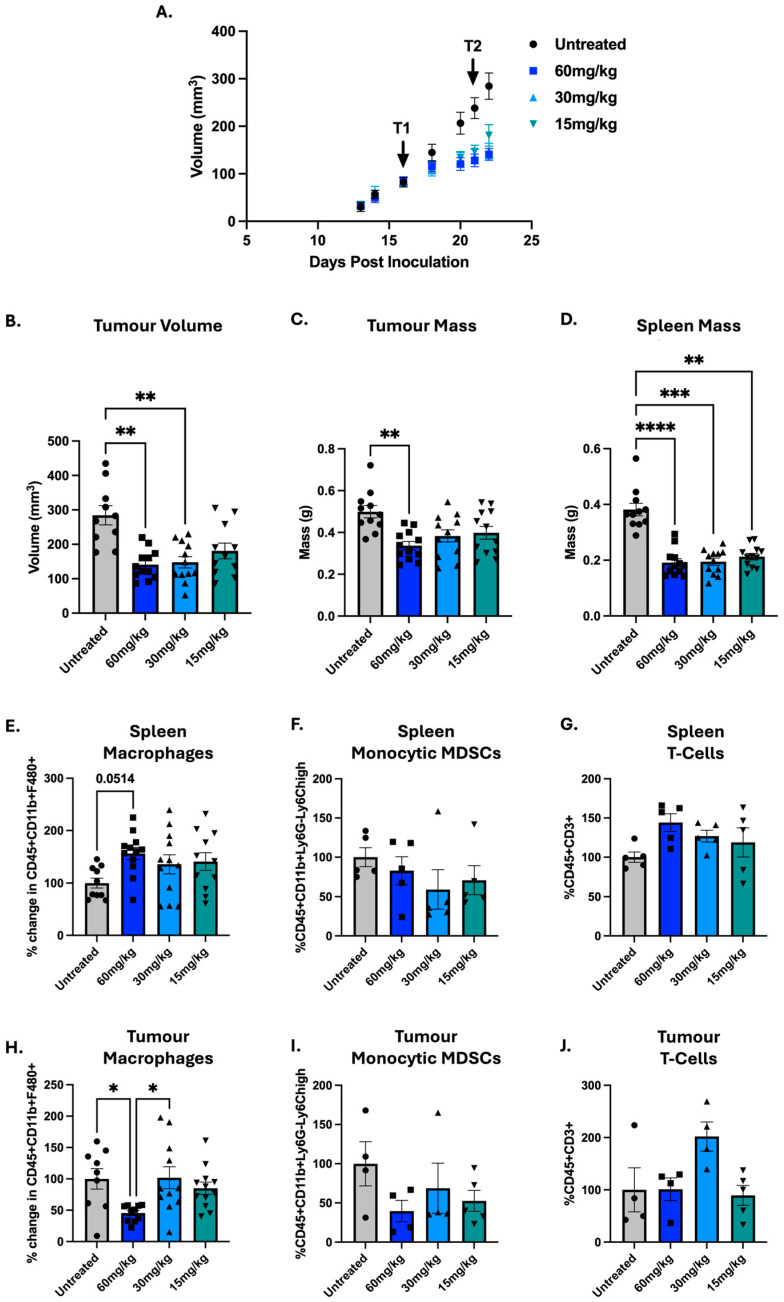
Dose-dependent effects of gemcitabine on tumour growth and immune cell populations in 4T1-bearing mice. 5 × 10^4^ 4T1 cells were inoculated into the fourth mammary fat pad of female BALB/c mice (Day 0). Mice were treated intraperitoneally with gemcitabine (GEM; 60, 30 or 15 mg/kg) on Days 16 (Treatment1 (T1)) and 21 (Treatment1 (T2)). Two independent experiments were performed. Results were normalised within each experiment to untreated mice and subsequently combined. (**A**) Tumour growth was monitored throughout the study with (**B**) final tumour volumes compared on Day 22. Tumours and spleens were harvested 24 h after the second treatment (Day 22). (**C**) Tumour and (**D**) spleen mass were measured. Flow cytometry was used to quantify immune populations in the (**E**–**G**) spleen and (**H**–**J**) tumour, including macrophages (CD45^+^CD11b^+^F4/80^+^), monocytic MDSCs (CD45^+^CD11b^+^Ly6G^−^Ly6C^high^) and T-cells (CD45^+^CD3^+^). Data are shown as mean ± SEM, with each point representing an individual mouse. The ROUT outlier test was applied (Q = 10%) and statistical significance was assessed using the nonparametric Kruskal–Wallis test with Dunn’s correction for multiple comparisons; * *p* < 0.05, ** *p* < 0.01, *** *p* < 0.001, **** *p* < 0.0001.

In mice, the lethal dose that kills 50% of mice (LD_50_) of GEM I.P. is reported to be 2000 mg/kg [32] and the maximum tolerated dose (MTD) for a single dose was 700 mg/kg [33]. In mice tumour studies, a single treatment of up to 400 mg/kg GEM has been used [34], with 60 mg/kg I.P. being common for 4T1 studies [19,20], which was effective in both reducing tumour volume and GR1^+^ MDSCs, described as pan-MDSCs from here on out [19]. To assess whether the observed immune effects were dose-dependent, we compared 60 mg/kg to 30 and 15 mg/kg in two independent studies, with data normalised as percent change within each experiment and combined where possible (Figure 2).

All three dosages were able to reduce tumour volume and mass in a similar manner; however, 15 mg/kg appears to be slightly less effective (Figure 2A–C). Next, the impact on immune populations was interrogated with changes observed in the T-cell, MDSC and macrophage populations as before (Figure 2E–J). Sixty mg/kg was the only dose that significantly decreased tumour macrophages (*p* = 0.0177), with a reduction of ~50% (Figure 2H). While all three dosages did not statistically significantly decrease M-MDSCs or increase T-cells, a trend is seen and could become significant with a larger sample size (as noted in our final combined analysis (Figure 4)).

### 3.3. I.P. Injection Can Be Used as a Surrogate for I.V. Injections

**Figure 3:**
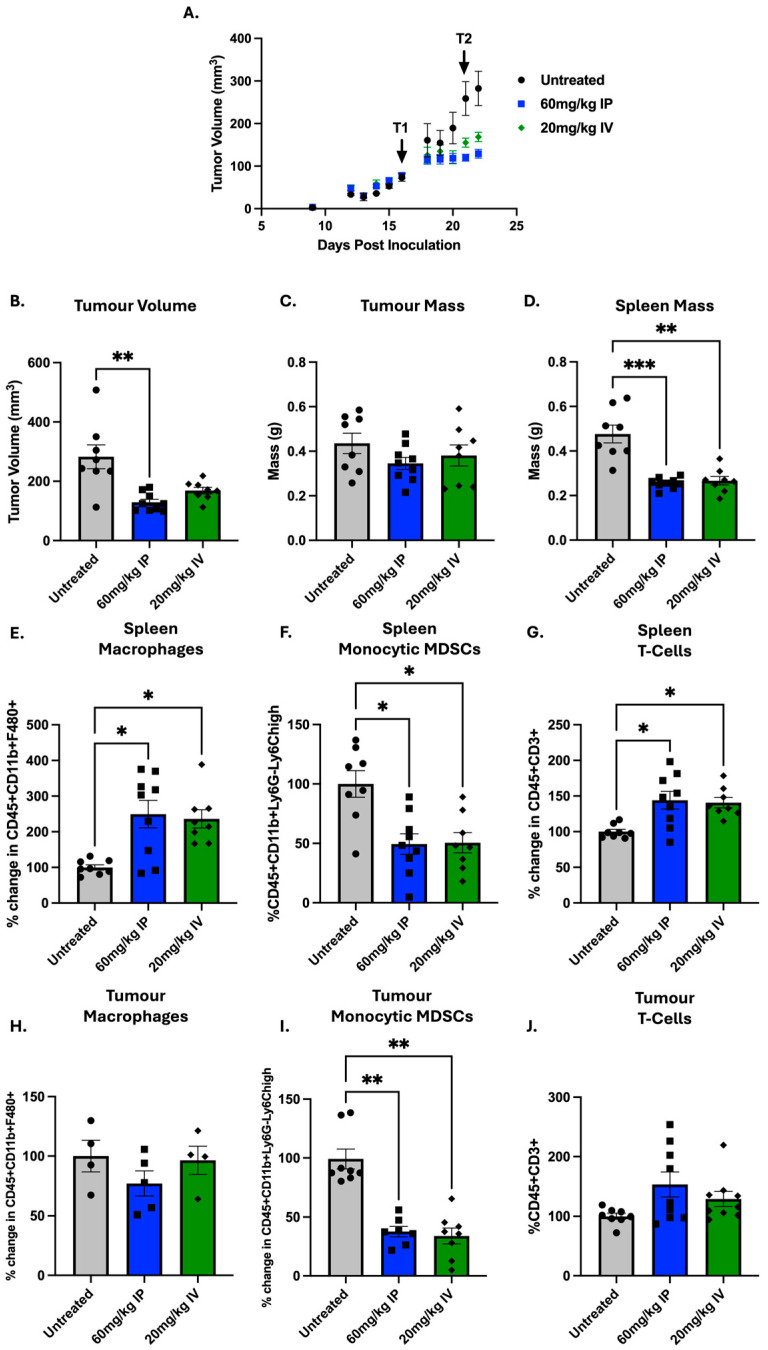
Differences in immune populations after treatment with I.P. or I.V. GEM in 4T1 tumour-bearing mice: 5 × 10^4^ 4T1 cells were inoculated into the fourth mammary fat pad of female BALB/c mice (Day 0). Mice were treated either intraperitoneally with 60 mg/kg GEM or intravenously with 20 mg/kg GEM on Days 16 (Treatment1 (T1)) and 21 (Treatment1 (T2)). Two independent experiments were performed. Results were normalised within each experiment to untreated mice and subsequently combined. (**A**) Tumour growth was monitored throughout the study with (**B**) final tumour volumes compared on Day 22. Tumours and spleens were harvested 24 h after the second treatment (Day 22). (**C**) Tumour and (**D**) spleen mass were measured. Flow cytometry was used to quantify immune populations in the (**E**–**G**) spleen and (**H**–**J**) tumour, including macrophages (CD45^+^CD11b^+^F4/80^+^), monocytic MDSCs (CD45^+^CD11b^+^Ly6G^−^Ly6C^high^) and T-cells (CD45^+^CD3^+^). Data are shown as mean ± SEM, with each point representing an individual mouse. The ROUT outlier test was applied (Q = 10%) and statistical significance was assessed using the nonparametric Kruskal–Wallis test with Dunn’s correction for multiple comparisons; * *p* < 0.05, ** *p* < 0.01, *** *p* < 0.001.

While I.P. injection is easy and reliable for in vivo models, intravenous (I.V.) administration is more clinically relevant and could possibly have a different effect systemically. Therefore, we compared 60 mg/kg I.P. to 20 mg/kg I.V. (dose guided by the published literature [35]) 24 h after the second treatment. Again, we preformed two completely independent studies and combined data when possible (Figure 3).

Both I.P. and I.V. reduced tumour volume to a similar extent; however, only 60 mg/kg saw a statistically significant reduction in volume (*p* = 0.0011), while neither saw a significant reduction in tumour mass. Both I.P. and I.V. significantly reduced spleen mass in comparison to untreated tumours (*p* = 0.0008 and 0.0050, respectively), which again correlates with a reduced tumour burden and a change in immune populations. A significant increase in splenic macrophages was seen in both I.P. (*p* = 0.0014) and I.V. (*p* = 0.0117) treatments in comparison to untreated tumours. However, while no significant changes in tumour macrophages were seen, there was a slight decrease observed in the I.P. treatment group. Both I.P. and I.V. groups significantly decreased splenic (*p* = 0.0234 and 0.0197, respectively) and tumour (*p* = 0.0048 and *p* = 0.0014, respectively) M-MDSCS, compared to the untreated group. In comparison to the untreated group, both I.P. and I.V. groups also significantly increased splenic T-cells (*p* = 0.01019, *p* = 0.0123, respectively) and increased tumour T-cells, although statistically insignificant.

### 3.4. Gemcitabine Is Effective in Reducing Pro-Tumourigenic Populations and Increasing T-Cells

**Figure 4:**
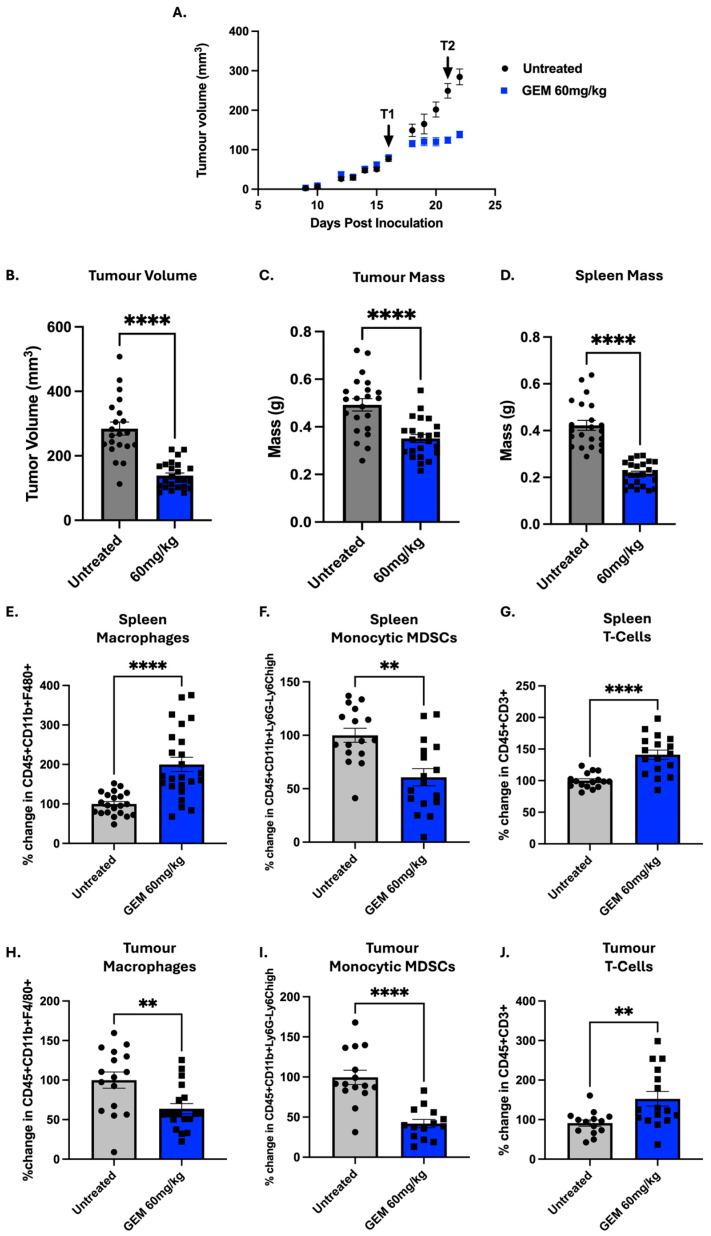
The immune profile of gemcitabine: 5 × 10^4^ 4T1 cells were inoculated into the fourth mammary fat pad of female BALB/c mice (Day 0). Mice were treated intraperitoneally with gemcitabine (GEM; 60 mg/kg) on Days 16 (Treatment1 (T1)) and 21 (Treatment1 (T2)). Five independent experiments were performed. Results were normalised within each experiment to untreated mice and subsequently combined. (**A**) Tumour growth was monitored throughout the study with (**B**) final tumour volumes compared on Day 22. Tumours and spleens were harvested 24 h after the second treatment (Day 22). (**C**) Tumour and (**D**) spleen mass were measured. Flow cytometry was used to quantify immune populations in the (**E**–**G**) spleen and (**H**–**J**) tumour, including macrophages (CD45^+^CD11b^+^F4/80^+^), monocytic MDSCs (CD45^+^CD11b^+^Ly6G^−^Ly6C^high^) and T-cells (CD45^+^CD3^+^). Data are shown as mean ± SEM, with each point representing an individual mouse. The ROUT outlier test was applied (Q = 10%) and statistical significance was assessed using nonparametric unpaired Mann–Whitney U-test ** *p* < 0.01, **** *p* < 0.0001.

In total, we conducted five independent in vivo studies (*n* = 45) investigating the immune role of GEM. While changes in immune populations were observed in each individual experiment, variation within groups (which is expected in such studies) meant some of these findings failed to reach significance. Therefore, to allow robust analysis and increase statistical power, all experiments with 60 mg/kg treated mice, where tissue was collected 24 h after treatment #2, were normalised to percent change within each experiment and combined. As seen in individual experiments, there was a significant increase in CD3^+^ T-cells both in the tumour (1.7 FC, *p* = 0.0045) and in the spleen (1.4 FC, *p* < 0.0001) when treated with GEM (Figure 4). However, there were no differences in splenic or tumour T-helper or cytotoxic T-cell abundance upon treatment (Figure S4C,D,G,H). Next, we saw a significant reduction in M-MDSCs in both the tumour (0.4 FC, *p* < 0.0001) and spleen (0.6 FC, *p* = 0.0016) following GEM treatment. While individual experiments did not reach statistical significance, after combining data, there was a small but significant increase in pan-MDSCs (GR1^+^) (1.1 FC, *p* = 0.0006) and granulocytic MDSCs (Ly6G^+^) (1.1 FC, *p* = 0.0089) in the tumour, as well as a decrease in granulocytic MDSCs (0.7 FC, *p* = 0.0061) in the spleen (Figure S4). As in the analysis of each separate experiment, there was a statistically significant decrease in tumour macrophages (0.6 FC, *p* = 0.0058), while a significant increase in spleen macrophages was seen (2.0 FC, *p <* 0.0001). Kitelinger et al.’s gating strategy was different for the macrophage population; therefore, we also analysed our data according to their gating strategy to allow direct comparison [20]. Using their gating strategy, we still saw a significant decrease in tumour macrophages (CD45^+^CD11b^+^F480^+^Ly6C^−^, 0.6 FC, *p* = 0.0021) and increase in splenic macrophages (3.1 FC, *p* < 0.0001, Figure S5A,C). We also used their gating for inflammatory monocytes CD45^+^CD11b^+^F480^+^Ly6C^+^), where we saw a significant decrease in both the tumour (0.4 FC, *p* < 0.0001) and spleen (0.6 FC, *p* = 0.0008, Figure S5B,D).

### 3.5. Transcriptional Changes Following Gemcitabine Treatment

**Figure 5:**
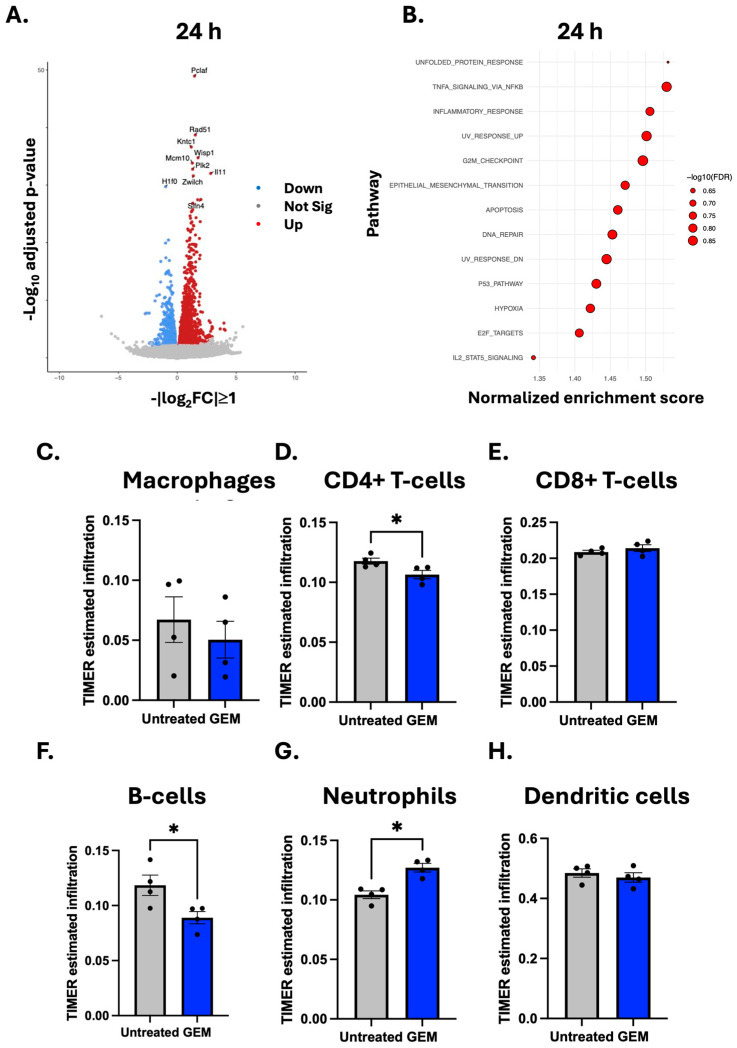
RNASeq analysis reveals changes in gene expression, immune signalling pathways and tumour-infiltrating immune cells in 4T1 tumours following gemcitabine treatment. (**A**) Volcano plot of RNASeq derived differentially expressed genes between 4T1 tumours harvested 24 h after the second dose of gemcitabine (GEM, 60 mg/kg) in comparison to untreated controls. Genes with a fold change (≥2) and an adjusted *p*-value (<0.05) are highlighted in red (upregulated) and blue (downregulated), with the 10 most significantly differential genes labelled. (**B**) Gene set enrichment analysis of GEM-treated tumours. The normalised enrichment scores (NES) and adjusted *p*-value for the top 13 significantly modulated pathways are displayed. TIMER-based estimates of infiltration levels for (**C**) macrophages, (**D**) CD4^+^ T-cells, (**E**) CD8^+^ T-cells, (**F**) B-cells, (**G**) neutrophils and (**H**) dendritic cells in untreated versus GEM-treated tumours. Data are shown as mean ± SEM, with each point representing an individual mouse. The ROUT outlier test was applied (Q = 10%) and statistical significance was assessed using nonparametric unpaired Mann–Whitney U-test: * *p* < 0.05.

Transcriptional analysis using RNASeq identified 370 upregulated and 75 downregulated genes following GEM, using a cut off of log2FC > ± 1, *adj.p* < 0.05 (Figure 5A), with top genes involved in DNA repair and cell cycle consistent with the DNA-damaging function of GEM. Interestingly, both pro- and anti-inflammatory genes were upregulated, including IL6 and IL10. Gene set enrichment analysis was used to identify key pathways, with 13 pathways significantly upregulated following GEM treatment (FDR < 0.25, Figure 5B), including apoptosis and DNA repair. Furthermore, the TNFα and inflammatory response pathways were also upregulated, not only demonstrating the cytotoxic effect of GEM but also the possible immune effect of GEM on the tumour. Of note, no pathways were significantly downregulated following GEM treatment (Figure 5B). Given the enrichment of immune-related pathways, deconvolution of the bulk RNASeq data was performed using TIMER estimates [24] for immune populations infiltration (Figure 5). Consistent with our flow cytometry data, a downregulation of macrophages was observed, although this was not significant (Figure 5C). TIMER also indicated no change in CD8^+^ T-cells (Figure 5E) or dendritic cells (Figure 5H). However, a significant reduction in CD4^+^ T-cells (*p* = 0.0286, Figure 5D) and B-cells (*p* = 0.0286, Figure 5F) with a significant increase in neutrophils (*p* = 0.0286, Figure 5G) was detected.

### 3.6. The Direct and Indirect in Vitro Effect of Gemcitabine on Macrophage Recruitment and Polarisation

The reduction in macrophages in the TME could be due to two major reasons: either (i) direct toxicity of GEM on macrophages or (ii) reduced recruitment of macrophages. While macrophages play a large role in 4T1 in vivo models, they only make up <15% of cells [36], making it difficult to accurately gate for subpopulations of macrophages without running high cell numbers per sample. Therefore, we pivoted to an in vitro approach utilising the monocyte cell line, THP1, which can be differentiated into a M_0_ phenotype (differentiated THP1 (D-THP1)) and further polarised towards M1- or M2-like states [37].

**Figure 6:**
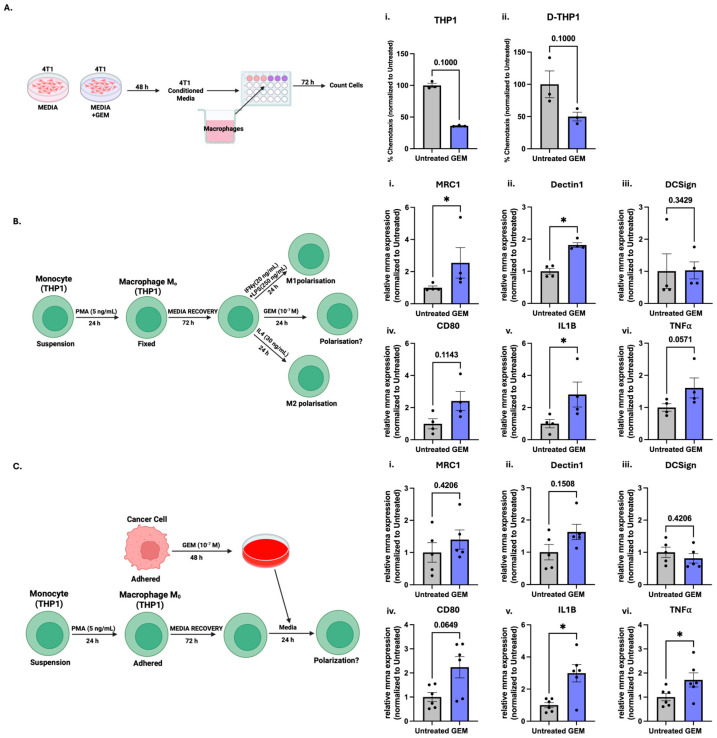
Gemcitabine-induced macrophage recruitment and polarisation: (**A**) 4T1 cells were treated with GEM (100 nM) for 48 h. (**i**) THP-1 monocytes (**ii**) differentiated D-THP-1 macrophages chemotaxis across a transwell towards the 4T1-conditioned media which was measured at 72 h. Data are presented as the percentage of migrated cells compared to the untreated control. (**B**) Differentiated macrophages (D-THP-1) were treated with GEM (100 nM) for 24 h or (**C**) conditioned media from 4T1 cells treated with GEM (100 nM) or control for 48hr. Gene expression of M1 and M2 polarisation markers was measured by qPCR. The graphs show the relative mRNA expression of M2 markers MRC1 (**i**), Dectin1 (**ii**) and DCSign (**iii**), and M1 markers CD80 (**iv**), IL1B (**v**) and TNFα (**vi**). Data are normalised to the untreated control. Data are shown as mean ± SEM, with each point representing an individual replicate. The ROUT outlier test was applied (Q = 10%) and statistical significance was assessed using nonparametric unpaired Mann–Whitney U-test: * *p* < 0.05.

To investigate the first possibility, we assayed cell viability by Resazurin following treatment observing that GEM had no impact on THP1 or D-THP1 cell viability (Figure S6A,B). Next, we investigated whether GEM was able to decrease macrophage recruitment. To do this, we first treated 4T1 cells with a range of concentrations of GEM, observing a classical dose response with an IC50 of 81 nM at 72 h (Figure S6C). We saw a similar dose response reduction in a clonogenic and spheroid model (Figure S6D–F). A concentration similar to the IC50 of 100 nM or 0.1 mM was selected for all subsequent studies. After treating 4T1 cells, the media was directly used in a transwell chemotaxis assay, where we observed a decrease in monocyte (THP1) and macrophage (D-THP1) recruitment towards GEM-treated conditioned media compared to control media (Figure 6A).

Next, we wanted to investigate any potential impact on macrophage polarisation, which GEM has the potential to do in at least two different ways. First, GEM can directly affect the macrophages. Alternatively, GEM can induce changes in the cancer epithelial cells that can lead to changes in secreted factors within the TME which can then impact macrophage polarisation indirectly. Therefore, two different approaches were utilised to model these scenarios. First using IFNγ + LPS or IL4 as positive controls [37], we demonstrated we could direct D-THP1 into a M1- or M2-like state, respectively (Figure S7). To study the direct effect of GEM on macrophage polarisation, D-THP1 cells were treated with 100 nM GEM for 24 h, mimicking the in vivo time point where the immune impact was greatest. The indirect effect was also studied, where 4T1 cells were treated with 100 nM GEM for 48 h and the media transferred to D-THP1 cells for 24 h. Following both approaches, we then assessed macrophage polarisation using the previously validated panel of genes. Interestingly, in both cases, both M1- and M2-like markers were increased following direct or indirect GEM treatment, indicating induction of a mixed phenotype suggestive of a complex immune response (Figure 6B,C).

## 4. Discussion

The TNBC TME is complex encompassing both pro- and anti-tumourigenic immune cell types. While there have been recent developments in immunotherapies, chemotherapy remains the backbone of standard of care treatment for TNBC, which, in first line, often includes a combination of 5-flourouracil, epirubicin/doxorubicin and cyclophosphamide (FEC), taxanes, and in a small population, platinum-based agents [3]. While most think that chemotherapy is universally immune-ablative, these cytotoxic agents have been shown to have a mixed role on the immune system such as cyclophosphamide decreasing T-regs but also increasing MDSCs, doxorubicin decreasing MDSCs and carboplatin having a limited to negative immune role [38]. However, GEM, used for the treatment of mTNBC, has shown the greatest potential as an immune modulator, reducing T-regs, MDSCs, as well as having a potential effect on macrophage polarisation [38,39,40,41]. While the role of chemotherapies as immune modulators has been investigated, there is high variability in dosage, number of treatment cycles, route of administration and time to analysis (i.e., when the tissues were collected after treatment). Given the highlighted potential of GEM, in this paper, we explore its pharmacodynamic immune properties including the temporal profile, minimum effective (immune) dose and route of administration for the treatment of TNBC. In total, we conducted five independent studies, analysing each individually. Finally, where possible, the data was normalised within each experiment before combining them, allowing robust statistical analysis to be performed.

It has been well reported that 4T1 tumour-bearing mice experience enlargement of the spleen, splenomegaly [19,25]. This has been shown by Younos et al., who correlated splenomegaly with increased abundance of MDSCs, dendritic cells and macrophages [25]. More specifically, the authors reported that the increase in MDSCs was inversely correlated with T-cell (CD3^+^) abundance [25]. We too report as tumour volume increases so does spleen mass, which is associated with an increase in MDSCs and decrease in T-cells in untreated mice.

First the kinetics of any immune modulation by GEM was considered. This knowledge is paramount when considering possible combination therapies to inform potential scheduling/sequencing to maximise clinical efficacy. For example, one study showed that the dose and the timing of paclitaxel in combination with a vaccine was crucial for the best results in a prostate cancer in vivo model [42]. They concluded that paclitaxel 2 days before the vaccine led to the highest infiltration of CD8^+^ cells and tumour regression, which matched other studies showing enhanced anti-tumour response with a 24–48 h pre-treatment with chemotherapy before vaccine [42].

Similarly to these and other studies [20], we found that GEM had the greatest immune impact at 24 h. This acute immune effect could be due to GEM’s very short half-life and its high clearance, where nearly all is eliminated via urine within 24 h in mice [43]. Furthermore, GEM was shown to accumulate in a pancreatic mouse orthoptic tumour within 2 h and is then subsequently cleared until there is no detection of GEM after 24 h [44]. Similarly in humans, GEM has a very short plasmid half-life of 5–20 min and 75% of GEM (dFdC) is converted to dFdU and excreted in the urine in the first 24 h [45]. We propose, therefore, GEM should be given before other immunotherapies, most likely within a window of a few hours and no more than 24 h apart.

Next, we investigated if GEM’s immune impact was dose dependent. The dose administered to a patient is crucial for optimal response and limited toxicity. There is a therapeutic range in which chemotherapy is effective in reducing tumour size while not having excess toxicity [46]. Classically, the maximum tolerated dosage (MTD), highest concentration without excess toxicity, of chemotherapy was given in cycles with long intervals to treat patients with cancer. However, the high dosage can have severe toxicity and the long intervals with no treatment can allow for drug-resistant cells to repopulate. Furthermore, when combining chemotherapy with a targeted therapy, only a small percent of combinations can use both the MTD for chemotherapy or recommended phase 2 dose (RP2D) for targeted therapy [47]. Therefore, it is essential to investigate the use of lower dosages of chemotherapy and how it impacts not only their cytotoxic role but also immunomodulatory role. In 2000, metronomic chemotherapy (MCT), where you treat with lower dosages without any drug-free periods, was introduced [48]. MCT can have benefits over using the historical MTD approach such as lower acute toxicity, overcoming chemotherapy resistance and can have immune benefits such as reducing T-regs [49]. In the TONIC clinical trial, metastatic TNBC patients received low-dose chemotherapy and results indicated that this regime may induce a more favourable pro-inflammatory tumour microenvironment and thus an increased response to PD-1 blockade [50]. Firstly, we saw that 30 and 60 mg/kg reduced tumour burden in a similar manner. However, we predict if it were left longer, the 15 mg/kg mice may have had a faster increase in tumour volume compared to 30 and 60 mg/kg. Therefore, we predict the minimum therapeutic dose to be above 15 mg/kg under the standing dosing regimen. However, we did not assess whether increasing the dosage frequency could allow for these lower doses to have an increased anti-tumour effect. On the other hand, we saw that 60 mg/kg was the lowest effective dose for immune modulation. Therefore, while MCT approach of GEM has its benefits, it remains unclear whether this approach hinders the desired immune response when combining with other therapies.

Many in vivo studies utilise I.P. delivery of therapeutics, including the two aforementioned 4T1 GEM studies, due to its ease of administration and low stress on the mice. While studies have shown drugs given I.P. have similar pharmacokinetics compared to when given via I.V. administration [51], we wanted to ensure GEM had similar cytotoxic and immune properties when given I.V., the clinically relevant route of administration. We compared 60 mg/kg I.P. to 20 mg/kg I.V. and showed that both are effective in reducing tumour volume and both change the same immune populations with no significant difference between them. We acknowledge that each route will have different pharmacodynamic properties upon administration and further investigation is needed to optimise dosing. However, for the sake of these studies, we can conclude that I.P. delivery of GEM can likely be used a surrogate for I.V. injections for in vivo models. This is beneficial when combining other therapies in which I.V. administration is necessary, limiting the strain on the mice as well as maintaining access to the veins.

Throughout our five studies, we saw three major immune populations change after treatment with GEM: T-cells, MDSCs and macrophages. Firstly, we saw that GEM significantly increased the abundance of overall T-cells (CD3^+^) both in the tumour and spleen. Higher abundance of tumour-infiltrating lymphocytes (TILs), which include T-cells and B-cells, have been correlated with better prognosis in breast cancer patients [52]. More specifically, studies have also shown that CD3^+^ expression is associated with more favourable outcomes in TNBC patients [53]. When looking into T-cell subtypes, it is well documented that increased CD8^+^ expression is associated with better survival in TNBC patients [53]. While the overall T-cell abundance increased in the spleen and tumour, we saw no significant change in either T-helper or cytotoxic T-cells abundance following GEM relative to untreated mice. While there were no significant changes, there was a small trend in decreasing tumour T-helper cells after GEM treatment. This is similar to what we saw via RNASeq deconvolution using TIMER, where no change in CD8^+^ infiltration and a significant decrease in CD4^+^ infiltration was seen. This could possibly be explained by the mixed upregulation of both pro- (IL6) and anti-inflammatory (IL10) cytokines seen in our analysis. This trend encourages further exploration into the T-helper subpopulation, T-regs, which GEM has shown to reduce in other cancer models [18]. Different to our data, Kitelinger et al. saw a significant decrease in tumour CD8^+^ T-cells and nonsignificant decrease in the CD4^+^ population in the tumour. Of note overall, CD3^+^ populations were not reported [20]. These differences could be attributed to the difference in number of GEM dosages, two in our studies vs. three in the other. This could mean that, in our study, while we have increased the overall T-cell population, they may not yet have been differentiated at the time of analysis. This could lead to the opportunity for an immunotherapy given at the appropriate time to differentiate these T-cells in cytotoxic T-cells for an increased anti-tumour effect.

Historically, MDSCs were classified solely by CD11b^+^GR1^+^ expression [54]. However, it has been found that there are two major subpopulations of MDSCs, monocytic (Ly6C^+^) and granulocytic (Ly6G^+^), with the classical GR1^+^ expression including both populations [54]. MDSCs can create an immunosuppressive TME by promoting M2-like polarisation and TH2 response. We saw a significant decrease in M-MDSCs both in the tumour and spleen. GEM (60 mg/kg) was also shown to reduce splenic and tumour M-MDSCs in E0771 tumour-bearing mice, an alternative TNBC model [55]. However, this decrease was only seen after a single treatment, while repeated treatment increased splenic M-MDSCs and had no impact on tumour M-MDSCs [55]. The reduction in M-MDSCs seen could be a possible explanation for the observed increase in T-cells. M-MDSCs are known to be able to suppress T-cell proliferation [56], thus, by reducing the abundance of MDSCs, an increase in CD3^+^ is possible [57]. Even though granulocytic MDSCs (G-MDSCs) are more abundant in 4T1 tumours [58], within each individual experiment, only small changes in the GR1^+^ or Ly6G^+^ populations was observed. Interestingly, when we combined our data, we saw a significant increase in tumour pan-MDSCs and G-MDSCs, while significantly decreasing splenic G-MDSCs. These results are similar to Le et al., who was first to show that GEM reduced MDSC abundance (GR1^+^) in the spleen, blood and bone marrow of 4T1 tumour-bearing mice [19]. Le et al., however, did not investigate the change in MDSCs in the tumour [19]. Again, similar to our results, Kitelinger et al. reported a significant decrease in G-MDSCs in the spleen and a significant increase in the tumour [20]. Although GEM increases G-MDSC abundance, which have immunosuppressive properties, we saw a reduction in M-MDSCs, which are more potent (per cell) in their immunosuppressive role [59,60]. Furthermore, unlike G-MDSCs, M-MDSCs are known to differentiate into immunosuppressive TAMs, making their reduction highly impactful [61].

Next, we observed a statistically significant decrease in the abundance of macrophages (F480^+^) in the tumour, while increasing the splenic macrophage population. The decrease in tumour macrophages is clinically significant as macrophages and, in particular, M2-like macrophages, have been correlated with a worse overall survival in TNBC patients [62]. While Kitelinger et al. did see a decrease in macrophages in the spleen they, however, did not show a decrease in the tumour macrophage population [20]. To better compare our results, we used the same gating strategy, but again we found that the splenic macrophage (F480^+^Ly6C^−^) population increased while the tumour population decreased. Again, this may be due to the differences in number of treatments between the studies and/or differences in immune populations due to the size of tumour.

Based on the inverse change in splenic and tumour macrophage abundance, we hypothesised that GEM was decreasing the recruitment of macrophages into the TME. This hypothesis was supported by our in vitro data where GEM-treated 4T1 cells were able to decrease recruitment of monocytes and macrophages. While we demonstrated that 4T1 had no cytotoxic effect on THP1/D-THP1 cells, thus not affecting the recruitment directly by decreasing cell viability, we cannot discount the possible immune impact residual GEM had on recruitment. Further studies should be performed with extra controls, including towards GEM alone, as well as removing the media from the 4T1 cells and incubating with fresh media before transferring to the transwell.

While we saw a clear reduction in recruitment, the impact on macrophage polarisation was less clear. Similarly to the mixed upregulation of cytokines, GEM increased both M1 and M2 macrophage markers. It has been shown macrophages can display a mixed polarisation state in TNBC [63], which GEM seems to also promote. Kitelinger et al. showed GEM increased Arg1^+^ expression, a marker for M2-like macrophages, in some monocyte populations, while decreasing Arg1^+^ expression in others [20]. Further studies are needed to validate this mixed upregulation on the protein level. However, we postulate that while GEM is unable to decrease M2-like macrophage or promote M1-like polarisation, it can reduce recruitment into the tumour, therefore negating the need to influence polarisation.

Deconvolution of the bulk RNASeq to interrogate immune populations also showed a decrease, albeit not significant, in the predicted macrophage population in the tumour. Unlike the other populations assessed by TIMER, the variability between replicates was large for the macrophage population, which could be leading to the data not being statistically significant. This variability could come from the overlapping of populations during deconvolution, as well as the inherent plasticity of macrophages. As we predict the macrophages may exhibit a mixed spectrum of polarisation states, traditional marker-based deconvolution may lead to estimation uncertainty. Furthermore, as TIMER is a human-based tool, the workflow includes ortholog mapping in order to analyse mouse data. However, the deconvolution method supports our flow data, indicating a decrease, albeit not significant, in the predicted macrophage population in the tumour. This comparable trend provides confidence that any potential mismatch at mapping did not impact the findings significantly, although to prove this, further validation by IHC or additional flow would be required. Of interest, the analysis highlighted a significant decrease in B-cell and increase in neutrophils. These results open a new window of exploration in which GEM may impact less abundant, however significant, subsets of immune cells within the TME.

## 5. Conclusions

Throughout this work, we explored the use of the chemotherapy agent, gemcitabine, as an immune modulator for the treatment of TNBC. We demonstrated that GEM’s effect was on three major immune populations, T-cells, MDCs and macrophages, and the effect was both transient and dose-dependent. GEM seems to create a mixed immune profile which caused no change in the CD4/CD8 ratio using flow cytometry or, seemingly, the M1/M2 ratio, based on preliminary studies. While we were able to show an increase in pro-inflammatory pathways and a reduction in macrophage recruitment, further studies are needed to understand how GEM is targeting M-MDSCs specifically. This work provides insight into anti-tumour activity of GEM beyond its role as an anti-metabolite and has set a foundation for the investigation of the combination of GEM with immunotherapies. While the temporal effects need to be validated in humans, from this data we suggest treating with GEM first, followed by immunotherapy within a 24 h window for the greatest possible therapeutic effect. While this study investigated many variables, we recognise that 4T1 is only one model of TNBC and others are needed to fully understand the immune role of GEM. Furthermore, combination studies are required to validate GEM’s ability to create an environment in which immunotherapies, such as checkpoint inhibitors, can have increased efficacy. Other current barriers include GEM currently being used only in second-line therapy rather than the early setting, as well as being given in combination with steroids to reduce side effects, which could dampen its immune effect. However, the role of GEM as both a cytotoxic agent and immune modulator for the treatment of TNBC is encouraging and should be further studied.

## Data Availability

The processed count matrix file is provided in the Supplementary Data. The raw data supporting the conclusions of this article will be made available by the authors on request.

## Supplementary Materials

The following supporting information can be downloaded at: https://www.mdpi.com/article/10.3390/cells14201604/s1, Figure S1: Example flow cytometry gating; Figure S2: Spleen Mass trends; Figure S3: Comparison of immune populations after three different treatments; Figure S4: Additional immune populations investigated using flow cytometry; Figure S5: Alternative flow cytometry gating based on previous literature; Figure S6: In-vitro cell viability assays; Figure S7: D-THP1 macrophage polarisation standards; Table S1: Real-time PCR primers; Table S2: Flow Antibodies.

## Abbreviations

The following abbreviations are used in this manuscript:

ARG1	Arginase 1
BC	breast cancer
CSF-1R	Colony stimulating factor receptor 1
DFS	disease-free survival
D-THP1	differentiated THP1 cells (macrophages)
ER	oestrogen receptor
GEM	gemcitabine
G-MDSCs	granulocytic myeloid-derived suppressor cells
HER2	human epidermal growth factor receptor 2
I.P.	intraperitoneal
I.V.	intravenous
IFNg	Interferon Gamma
IL4	Interleukin 4
iNOS	inducible Nitric Oxide Synthase
MDSC	myeloid-derived suppressor cells
M-MDSCs	monocytic myeloid-derived suppressor cells
OS	Overall Survival
PD-1	programmed cell death protein 1
PD-L1	programmed death ligand 1
PR	progesterone receptor
ROS	Reactive Oxygen Species
TAMs	tumour associated macrophages
TILs	tumour infiltrating lymphocytes
TME	tumour microenvironment
TNBC	triple negative breast cancer
TNFα	Tumour Necrosis Factor Alpha
T-regs	Regulatory T-cells
