# Supplementary material for: The Immunomodulatory Role of Gemcitabine in Triple Negative Breast Cancer
Source: Cells. 2025 Oct 16;14(20):1604. doi: 10.3390/cells14201604 (PMC12564518; doi:10.3390/cells14201604)
Supplement: Supplementary file 1 [file cells-14-01604-s001.zip › cells-3881540-supplementary.pdf]

Supplementary Table 1: Real-time PCR primers

Gene	Forward	Reverse
β-actin	TCC TCC CTG GAG AAG AGC TA	CGT GGA TGC CAC AGG ACT
CD80	GGG AAA TGT CGC CTC TCT GA	GTG GAT TTA GTT TCA CAG CTT G
DC Sign	TCA AGC AGT ATT GGA ACA GAG GA	CAG GAG GCT GCG GAC TTT TT
Dectin1	AAC CAC AGC TAC CCA AGA AAA C	GGG CAC ACT ACA CAG TTG GTC
IL1B	GTG GCA ATG AGG ATG ACT TGT TC	TAG TGG TGG TCG GAG ATT CGT A
MRC1	CAG CGC TTG TGA TCT TCA TT	TAC CCC TGC TCC TGG TTT TT
TNFa	CTG CTG CAC TTT GGA GTG AT	AGA TGA TCT GAC TGC CTG GG

Supplementary Table 2: Flow Antibodies		
**Target**	**Antibody Clone**	**Supplier**
Fc Block	S17011E	BioLegend
Live/Dead	Zombie Aqua Dye	BioLegend
CD45+	BUV395 (30-F11)	BD
CD11b+	BUV563 (M1/70)	BD
F480+	FITC (QA17A29)	BioLegend
F480+	Alexa Fluor 488 (BM8)	Invitrogen
GR1+	BUV805 (RB6-8C5)	BD
Ly6G+	BUV615 (1A8)	BD
Ly6C+	PerCP/Cy-5.5 (HK1.4)	BioLegend
CD3+	BUV496 (17A2)	BD
CD4+	Spark Violet 423 (RM4-5)	BioLegend
CD8+	BV650 (53-6.7)	BD

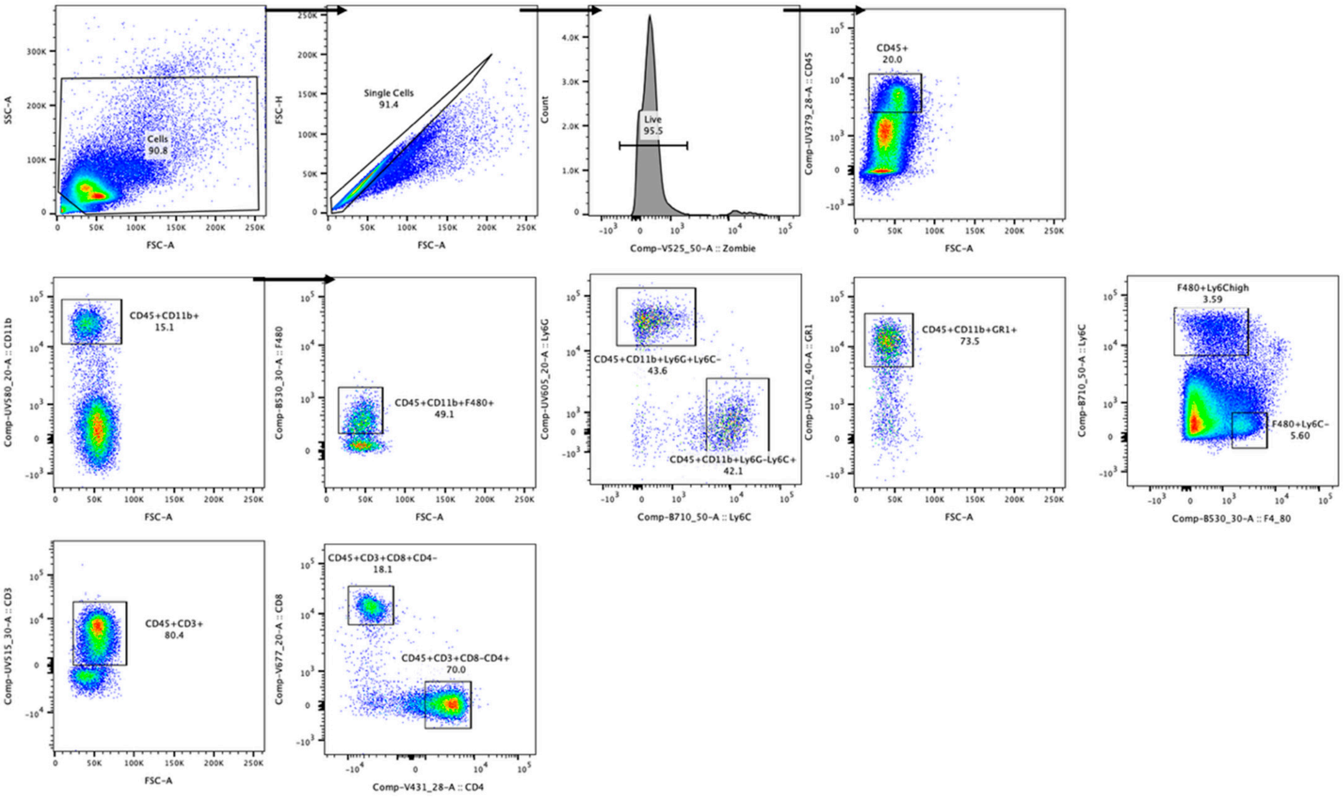

Supplementary Figure 1: Example flow cytometry gating. 5x10^4 4T1 cells were inoculated into the 4th mammary fat pad of female Balb/C mice (Day 0). Mice were treated with GEM. Spleen and tumour were collected. Flow cytometry was performed and representative gating for each immune population is shown. Analysis was done using FlowJo.

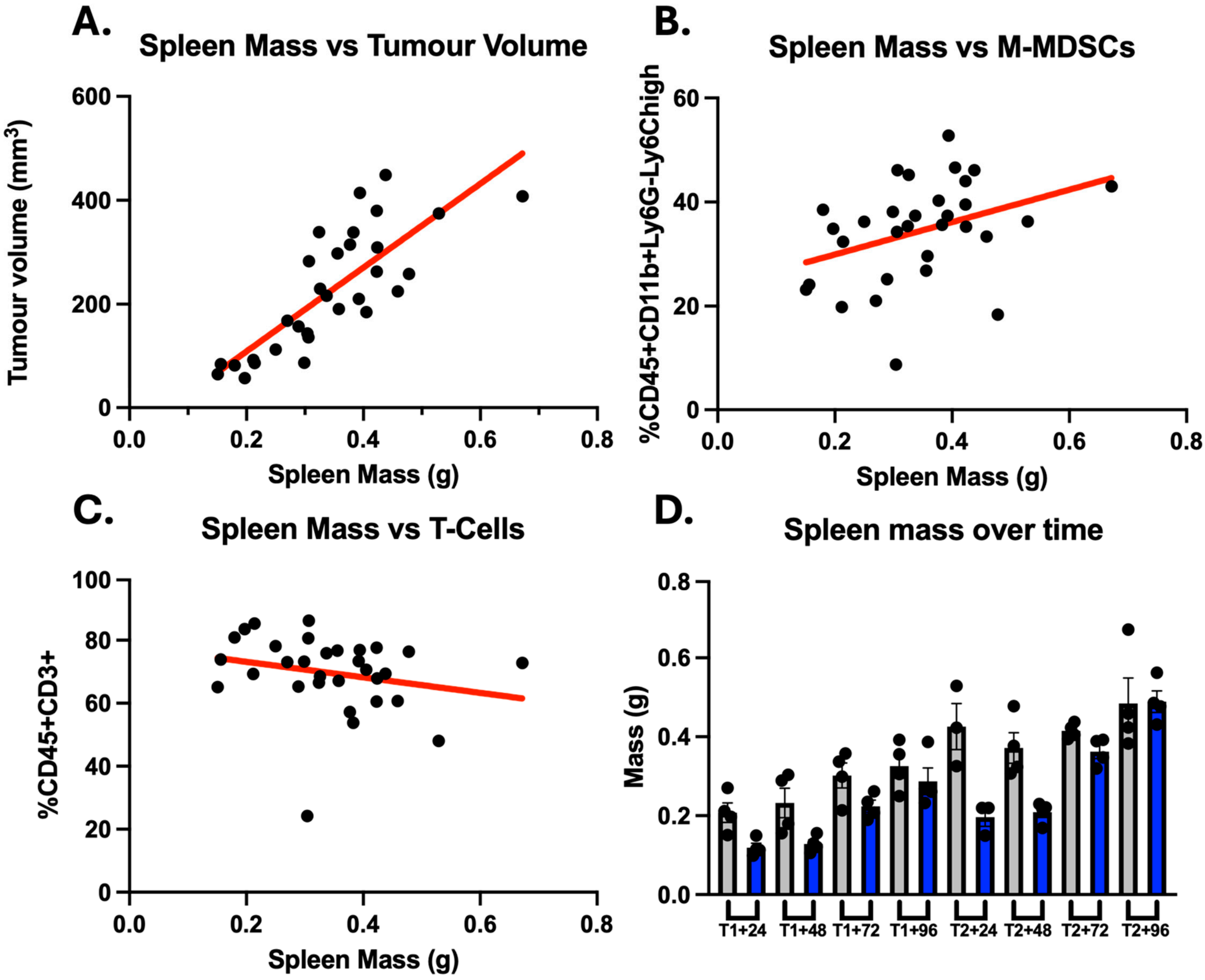

Supplementary Figure 2: Spleen Mass trends. 5x10^4 4T1 cells were inoculated into the fourth mammary fat pad of female BALB/c mice (Day 0). Mice were treated twice with 60mg/kg GEM on days 16 (T1) and 21 (T2) after cell inoculation. Tumour growth was monitored throughout the study. Tumours and spleen were collected 24-96 hours after treatment 1 and 2 (T1/2+24/48/72/96) and analysed via flow cytometry. Untreated mice trends were analysed for (A) Spleen mass vs tumour volume (B) Spleen mass vs M-MDSC (CD45+CD11b+Ly6G-Ly6Chigh) abundance and (C) Spleen mass vs T-cell (CD45+CD3+) abundance. (D) Change of spleen mass over time.

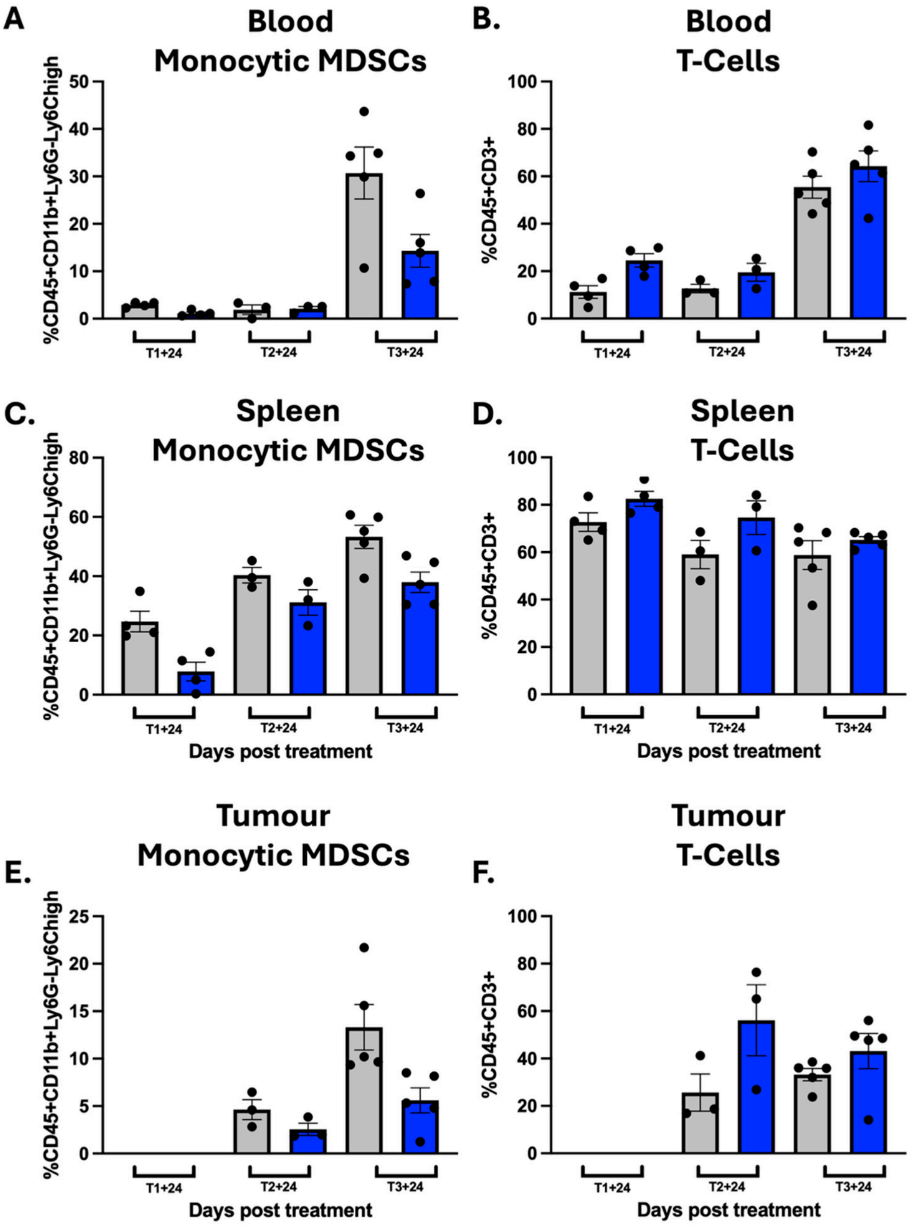

Supplementary Figure 3: Comparison of immune populations after three different treatments. 5x10^4 4T1 cells were inoculated into the fourth mammary fat pad of female BALB/c mice (Day 0). Mice were treated intraperitoneally with gemcitabine (GEM; 60 mg/kg) on Days 16 (T1) and 21(T2). Flow cytometry was used to quantify immune populations in the (A-B) blood, (C-D) spleen and (E-F tumour, including monocytic MDSCs ($CD45^+CD11b^+Ly6G^-Ly6C$high) and T-cells ($CD45^+CD3^+$). Data are shown as mean ± SEM with each point representing an individual mouse.

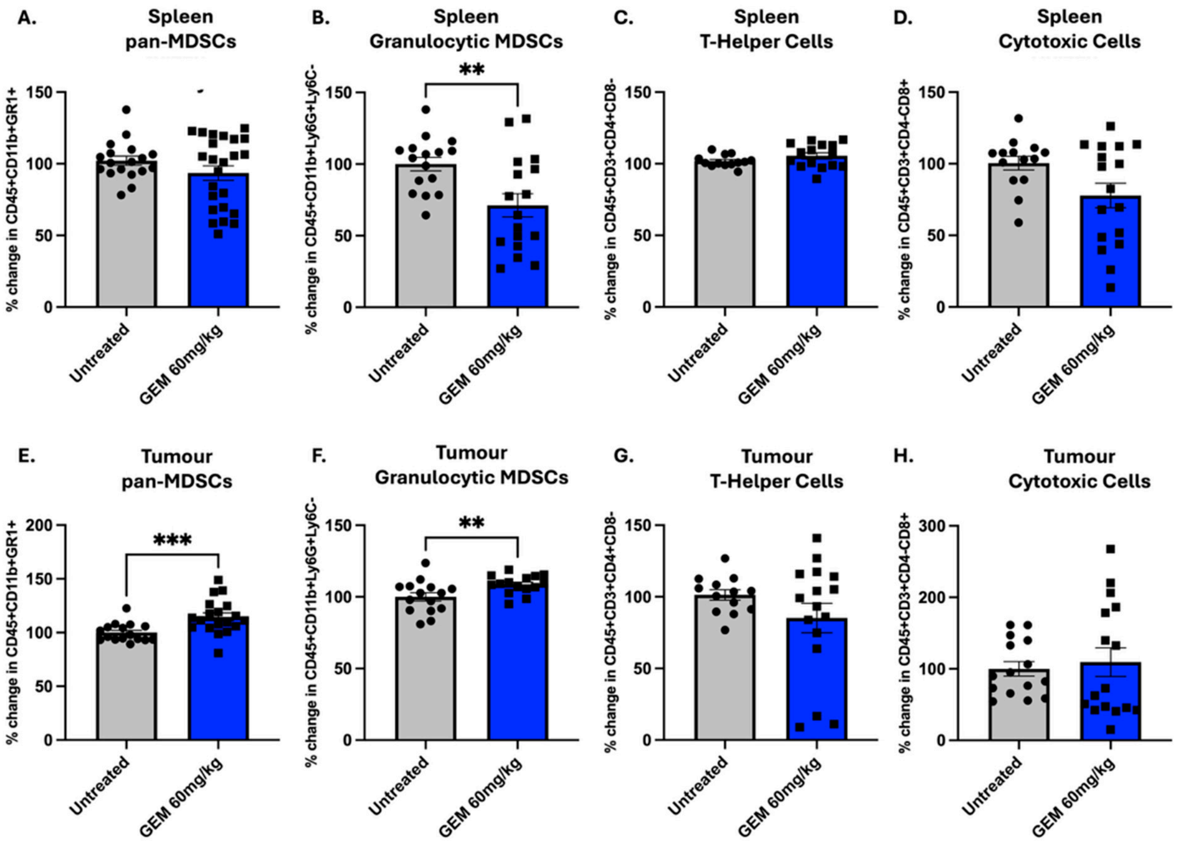

Supplementary Figure 4: Additional immune populations investigated using flow cytometry. $5x10^4$ 4T1 cells were inoculated into the fourth mammary fat pad of female BALB/c mice (Day 0). Mice were treated intraperitoneally with gemcitabine (GEM; 60 mg/kg) on Days 16 (T1) and 21(T2). Five independent experiments were performed. Results were normalised within each experiment to untreated mice and subsequently combined. Flow cytometry was used to quantify immune populations in the (A-D) spleen and (E-H) tumour, including pan-MDSCs ($CD45^+CD11b^+GR1+$), G-MDSCs (CD45+CD11b+Ly6G+Ly6C-), T-helper cells (CD45+CD3+CD4+CD8-) and cytotoxic T-cells (CD45+CD3+CD4-CD8+). Data are shown as mean ± SEM with each point representing an individual mouse. The ROUT outlier test was applied (Q = 10%) and statistical significance was assessed using nonparametric unpaired Mann-Whitney t-test *$p<0.05$, **$p<0.01$, ***$p<0.001$, ****$p<0.0001$

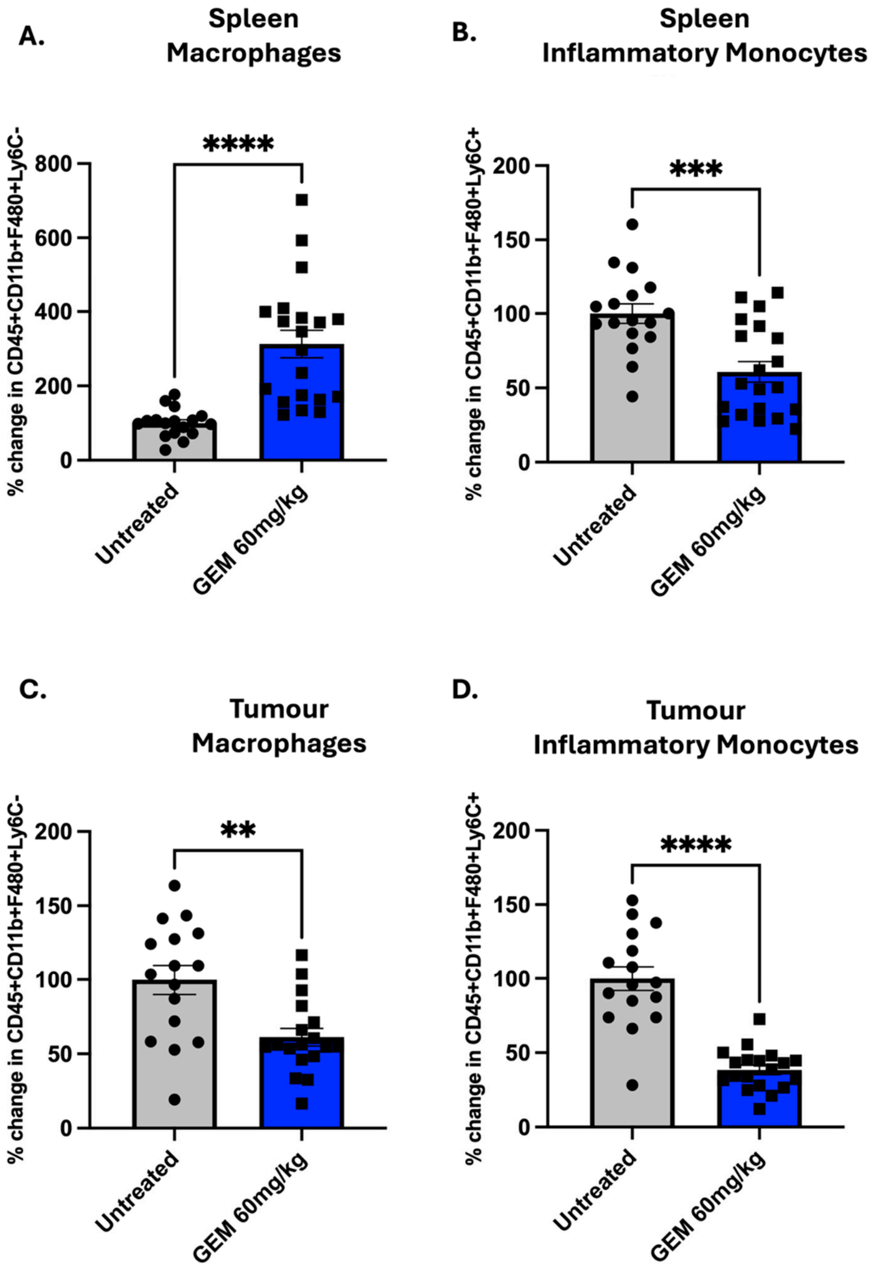

Supplementary Figure 5: Alternative flow cytometry gating based on previous literature. $5x10^4$ 4T1 cells were inoculated into the fourth mammary fat pad of female BALB/c mice (Day 0). Mice were treated intraperitoneally with gemcitabine (GEM; 60 mg/kg) on Days 16 (T1) and 21 (T2). Five independent experiments were performed. Results were normalised within each experiment to untreated mice and subsequently combined. Flow cytometry was used to quantify immune populations in the (A-B) spleen and (C-D) tumour, including macrophages ($CD45^+CD11b^+F4/80^+Ly6C-$) and inflammatory monocytes (CD45+CD11b+F480+ly6C+). Data are shown as mean ± SEM with each point representing an individual mouse. The ROUT outlier test was applied (Q = 10%) and statistical significance was assessed using nonparametric unpaired Mann-Whitney t-test *p<0.05, **p<0.01, ***p<0.001, ****p<0.0001

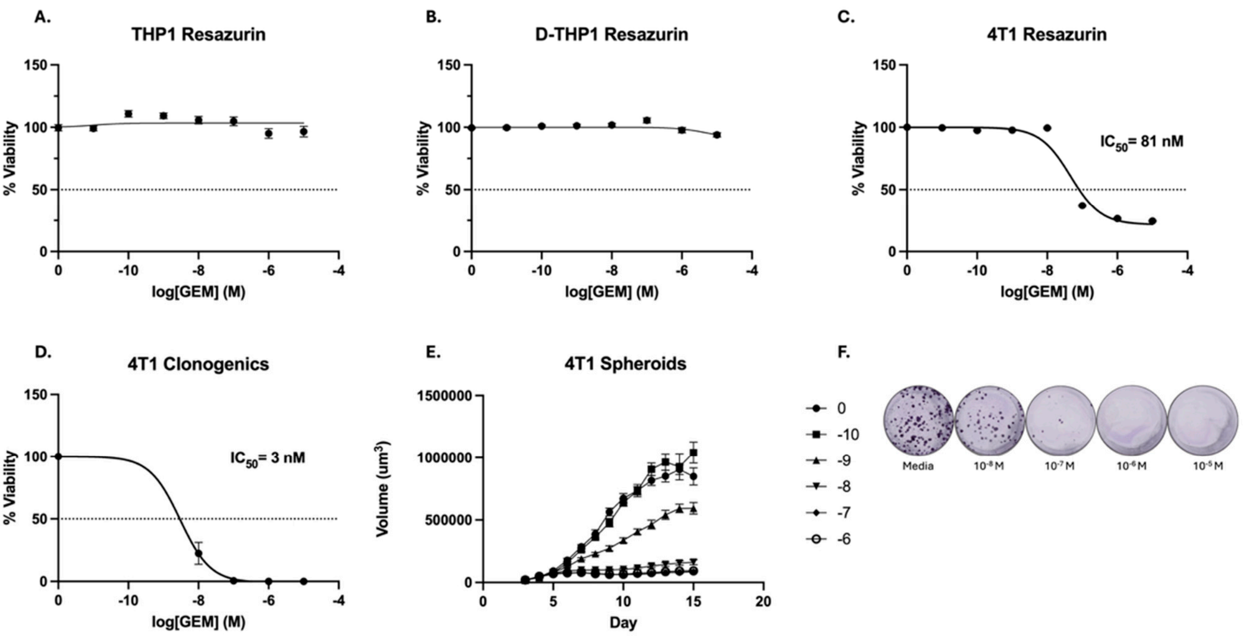

Supplementary Figure 6: In-vitro cell viability assays. (A) 4 x 10^4 THP1 cells, (B) 7.5 x 10^5 D-THP1 cells or (C) 4 x 10^4 4T1 cells were seeded in a 24-well dish and the next day treated with a range of concentrations of GEM for 72 hrs before cell viability was assayed using Resazurin. Dose response curves were plotted and IC50 calculated where possible (D) 4 x 10^4 4T1 cells were seeded in a 24-well plate and the next day treated with GEM for 24 hrs. Cells were lifted and 300 cells were seeded into a 6-well dish and left for 10 days. Cells were stained with crystal violet. (E) 500 4T1 cells were seeded with Matrigel in a 96-well round bottom low-attachment plate. Spheroids were treated with GEM on day 4 and volume was measured using a plate imager daily for 15 days. F) A representative stained clonogenic plate.

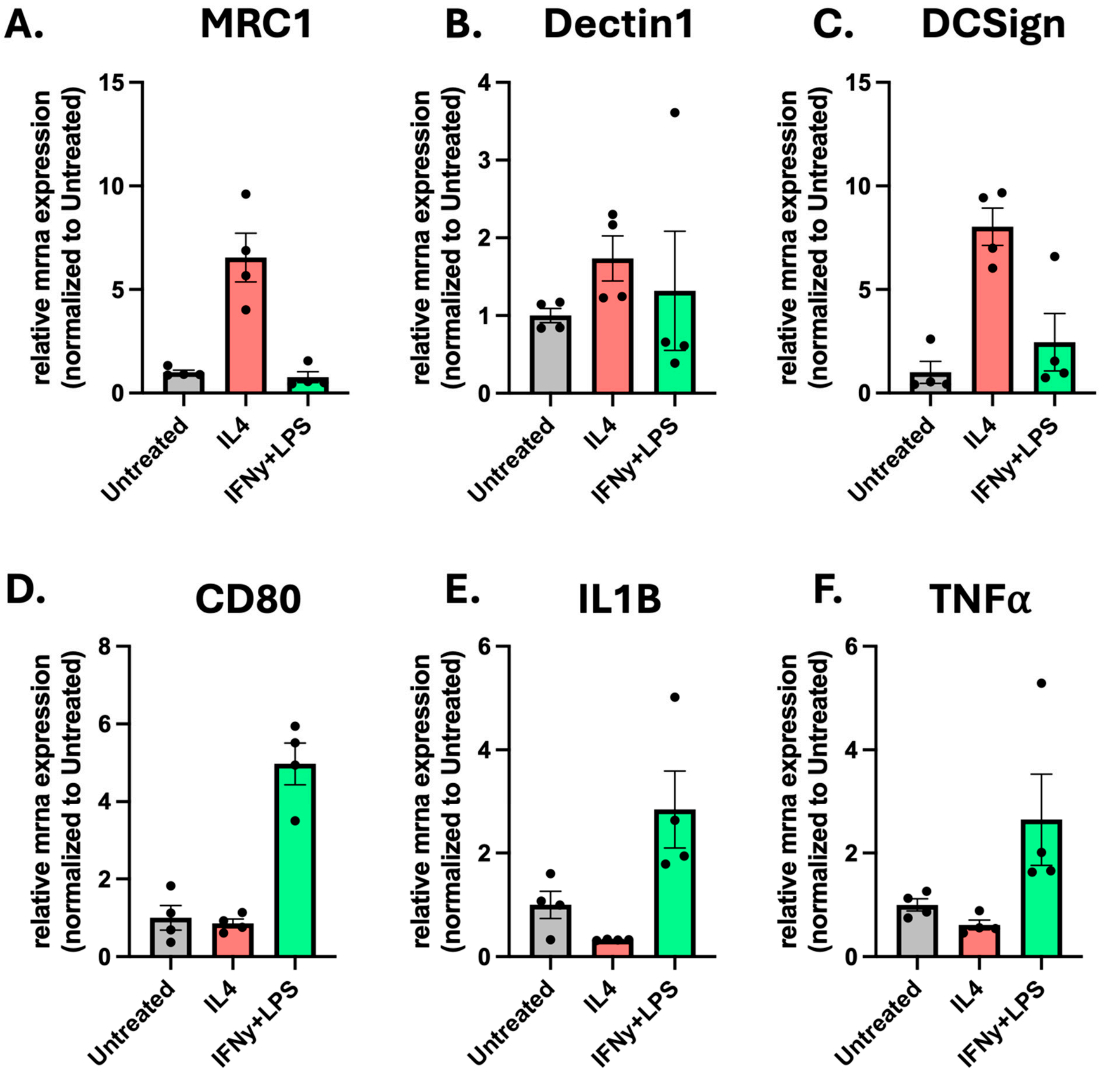

Supplementary Figure 7: D-THP1 macrophage polarisation standards. Differentiated macrophages (D-THP-1) were either untreated or treated with IL4 (30ng/mL) or IFNγ (20 ng/mL) + LPS (250 ng/mL) for 24 hrs. Gene expression of M1 and M2 polarization markers was measured by qPCR. The graphs show the relative mRNA expression of M2 markers (A) MRC1, (B) Dectin1 and (C) DCSign and M1 markers (D) CD80, (E) IL1B and (F) TNFα. Data are normalized to the untreated control. Data are shown as mean ± SEM with each point representing an individual replicate
